# The value of ultrasonography in predicting arthritis in auto-antibody positive arthralgia patients: a prospective cohort study

**DOI:** 10.1186/ar3028

**Published:** 2010-05-20

**Authors:** Lotte A van de Stadt, Wouter H Bos, Marlies Meursinge Reynders, Helen Wieringa, Franktien Turkstra, Conny J van der Laken, Dirkjan van Schaardenburg

**Affiliations:** 1 Rheumatology, Jan van Breemen Institute, Dr Jan van Breemenstraat 2, 1056 AB, Amsterdam, The Netherlands; 2 Radiology, Jan van Breemen Institute, Dr Jan van Breemenstraat 2, 1056 AB, Amsterdam, The Netherlands; 3 Rheumatology, VU Medical Centre, De Boelelaan 1118, 1081 HZ, Amsterdam, The Netherlands

## Abstract

**Introduction:**

Ultrasonography (US) has better sensitivity than clinical evaluation for the detection of synovitis in early rheumatoid arthritis (RA). Patients presenting with arthralgia and a positive anti-citrullinated protein antibodies (ACPA) and/or Rheumatoid Factor (IgM-RF) status are at risk for developing RA. In the present study, US utility and predictive properties in arthralgia patients at risk for the development of arthritis were studied.

**Methods:**

192 arthralgia patients with ACPA and/or IgM-RF were included. Absence of clinical arthritis was confirmed by two physicians. US was performed by one of two trained radiologists of any painful joint, and of adjacent and contralateral joints. Joint effusion, synovitis and power Doppler (PD) signal in the synovial membrane of the joints and tenosynovitis adjacent to the joint were evaluated and classified on a 4-grade semi-quantitative scale. Grade 2-3 joint effusion, synovitis, tenosynovitis and grade 1-3 Power Doppler signal were classified as abnormal.

**Results:**

Forty-five patients (23%) developed arthritis after a mean of 11 months. Inter-observer reliability for synovitis and PD was moderate (kappa 0.46, and 0.56, respectively) and for joint effusion low (kappa 0.23). The prevalence of tenosynovitis was too low to calculate representative kappa values. At joint level, a significant association was found between US abnormalities and arthritis development in that joint for joint effusion, synovitis and PD. At patient level, a trend was seen towards more arthritis development in patients who had US abnormalities for joint effusion, synovitis, PD and tenosynovitis.

**Conclusions:**

US abnormalities were associated with arthritis development at joint level, although this association did not reach statistical significance at patient level. US could potentially be used as a diagnostic tool for subclinical arthritis in seropositive arthralgia patients. However, further research is necessary to improve test characteristics.

## Introduction

Rheumatoid arthritis (RA) is a chronic inflammatory disease resulting in joint damage. The presence of auto-antibodies such as anti-citrullinated protein antibodies (ACPA) and/or immunoglobulin M-rheumatoid factor (IgM-RF) is a characteristic finding in RA. These auto-antibodies can often be detected years before the onset of clinical disease and can predict the development of arthritis [1-3]. Concomitant arthralgia appears to increase the risk of developing arthritis even further [3] and thus seropositive arthralgia patients could be considered as preclinical RA patients. This phase of the disease is of interest now that evidence supports very early intervention in RA [4]. Treating even earlier in the preclinical phase may prevent progression to RA. However, a substantial part of seropositive arthralgia patients does not develop arthritis [3] and it remains a challenge to separate true preclinical RA patients from plain arthralgia patients.

Ultrasonography (US) is a promising tool for use in the diagnosis of arthritis because it is relatively cheap, multiple joints can be examined in a short period of time and bone structure as well as soft tissue can be examined. Moreover, US has a better sensitivity than physical examination in the detection of synovitis in early arthritis and RA [5]. In a recent study of patients with morning stiffness of at least one hour with or without clinical arthritis, the presence of US abnormalities in hand joints increased the probability of later inflammatory arthritis, although only in the subgroup of seronegative patients. All seropositive patients developed arthritis and thus there was no additional value of US in these patients [6]. The present study concerns seropositive arthralgia patients alone, of whom a substantial part did not develop arthritis [3]. US utility and predictive properties were studied in this cohort.

## Materials and methods

### Study population

Between August 2004 and August 2008, arthralgia patients with a positive ACPA and/or IgM-RF status were recruited at rheumatology clinics in the Amsterdam area of the Netherlands after referral by a general practitioner. Patients without arthritis, but with a positive ACPA and/or IgM-RF status were referred for inclusion in the present study. Arthralgia was defined as non-traumatic pain in any joint. Absence of arthritis was independently confirmed by physical examination of 44 joints [7] by a trained medical doctor (WB or LAS) and a senior rheumatologist (DS). The latter was blinded for the patient's history and laboratory results. ACPA and/or IgM-RF were confirmed in a second sample. Patients with arthritis as revealed by chart review or baseline physical examination, a negative ACPA and IgM-RF status on second analysis, previous treatment with a disease-modifying antirheumatic drug (DMARD) or recent glucocorticoid treatment (<3 months) were excluded. In total, 192 arthralgia patients (72% female, mean ± standard deviation (SD) age 47.6 ± 11 years) with a positive ACPA and/or IgM-RF status were included in the present study. Of these patients, 70 were also included in a randomized placebo-controlled trial studying the effects of intramuscular dexamethasone on arthritis development. As dexamethasone did not delay or prevent arthritis [8] these patients were considered suitable for the present analysis.

At baseline, medical history, details of joint complaints and the number of tender joints at physical examination of 53 joints were recorded [9]. During yearly follow-up visits, development of arthritis in any of 44 joints [7] was confirmed by two investigators (WB or LAS and DS). Extra visits were planned if arthritis developed. Median follow up was 26 months (range 6 to 54 months).

Nine healthy controls (7 women and 2 men; mean age 47.3 years, range 36 to 58 years) without signs or symptoms of arthritis as confirmed by clinical examination of 44 joints [9] and with a negative ACPA and IgM-RF status were also studied.

This study was approved by the local ethics committee and informed consent was given by all patients prior to inclusion.

### Ultrasonography

US was performed within a median of three weeks (interquartile range (IQR) one to five weeks) after the first visit. If present, tender joints at physical examination were scanned, otherwise joints that were painful by history were scanned. Furthermore, for proximal interphalangeal (PIP), metacarpal phalangeal (MCP) and metatarsophalangeal (MTP) joints the directly adjacent joints in the same joint group as the painful joints were scanned, for example, in the case of a painful MCP3, MCP2 to 4 were scanned. US was also performed on the contralateral joints selected in this way. (As some patients had bilateral joint complaints, contralateral joints could include painful joints.) Joints were scored on a four-grade semiquantitative scale for joint effusion, synovitis, tenosynovitis and power Doppler signal, as described before and explained here [10].

Joint effusion was defined as a compressible anechoic intracapsular area and scored as follows: 0 = no effusion, 1 = minimal amount of joint effusion, 2 = moderate amount of joint effusion (without distension of the joint capsule) and 3 = extensive amount of joint effusion (with distension of the joint capsule). Synovitis was defined as a noncompressible hypoechoic intracapsular area (synovial thickening) and scored as follows: 0 = no synovial thickening, 1 = minimal synovial thickening (filling the angle between the periarticular bones, without bulging over the line linking tops of the bones), 2 = synovial thickening bulging over the line linking tops of the periarticular bones but without extension along the bone diaphysis, 3 = synovial thickening bulging over the line linking tops of the periarticular bones and with extension to at least one of the bone diaphyses. Power Doppler signal was used to display flow signal in the synovium and scored as follows: 0 = no flow in the synovium, 1 = single vessel signals, 2 = confluent vessel signals in less than half of the area of the synovium, 3 = vessel signals in more than half of the area of the synovium [10]. Tenosynovitis was defined as hypoechoic or anechoic thickened tissue with or without fluid within the tendon sheath and scored as follows: 0 = no thickened tissue, 1 = minimal thickening, 2 = moderate thickening, 3 = extensive thickening.

In the healthy controls, either MCP joint 2 to 4 and PIP joint 2 and 3 (n = 3), MCP joint 3 to 5 and PIP joint 4 and 5 (n = 3), or MCP joint 2 to 4 and the wrist joint (n = 3) were scanned. US was performed bilaterally.

In this study, grades 2 to 3 of joint effusion, synovitis and tenosynovitis were regarded as abnormal, and grades 1 to 3 power Doppler signal was regarded as abnormal [10,11].

All scans were performed with the Acuson Antares ultrasound system, premium edition (Siemens, Malvern, PA, USA) using linear array transducers VF 13-5 SP for finger and toe joints, (operating at 11.43 MHz for greyscale and 8.9 MHz for PD) and VF 13-5 for larger joints (operating at 11.43 MHz for greyscale and 7.3 MHz for PD), according to the manufacturer's criteria. All joints were scanned in the longitudinal plane from the most lateral to most medial site and in the transverse plane from the proximal to distal site of the joint.

The US examinations were performed by two independent investigators (HW and MMR), both radiologists with extensive experience in musculoskeletal US. The investigators were blinded for clinical data. Healthy controls were referred as if they were arthralgia patients. Prior to the study, consensus was reached between the investigators about scanning technique, pathology definitions and scoring.

Interobserver reliability was evaluated by scanning 148 joints of 14 patients, including 7 patients fulfilling ACR criteria of RA [12], successively by both investigators, who were blinded for each other's findings and all other study data.

### Laboratory investigations

ACPA and IgM-RF levels were determined at baseline by second-generation anti-cyclic citrullinated peptide (anti-CCP) ELISA (Axis Shield, Dundee, UK) and in-house ELISA as previously described [1].

### Statistical analysis

Data evaluation and statistical analysis were performed with SPSS version 16.0 software (SPSS Inc., Chicago, IL, USA). Continuous data with Gaussian distribution were summarized with mean and SD. Non-normally distributed data were summarized with median and IQR. Categorical data were analyzed by Chi-square test and results were expressed as odds ratio (OR) with 95% confidence interval (CI). Inter-observer agreement was calculated by quadratic weighted kappa-statistics for the ordinal data. After dichotomization of ordinal data as described above, regular kappa values and overall agreement were calculated.

## Results

### Arthritis development

**Figure 1:**
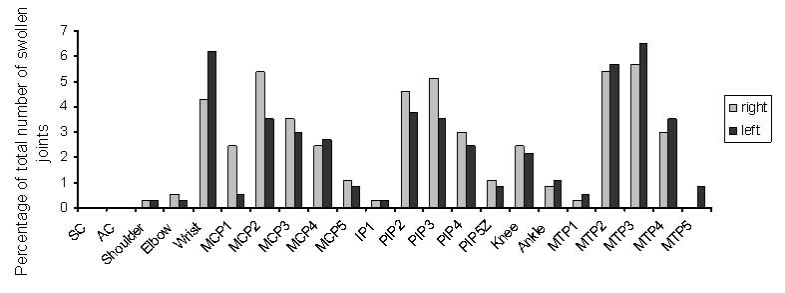
**Arthritis development of individual joints**. During yearly follow-up visits, development of arthritis in any of 44 joints was independently confirmed by two investigators. The percentage of all arthritic joints in which arthritis developed in that particular joint is shown. Grey bars represent the right sided joints, black bars the left sided joints. AC, acromioclavicular; IP, interphalangeal; MCP, metacarpal phalangeal; MTP, metatarsophalangeal; PIP, proximal interphalangeal; SC, sternoclavicular.

**Figure 2:**
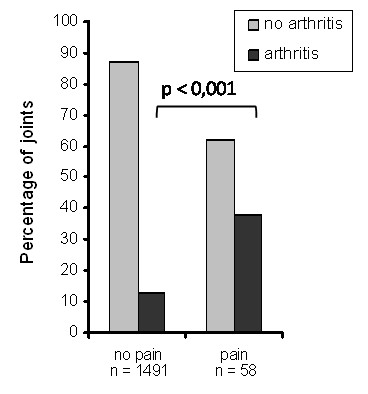
**Association of pain at baseline with arthritis development in joints of patients that developed arthritis**. At baseline 53 joints per patient were scored for the presence of pain at physical examination. At the time of arthritis development 44 joints were scored for the presence of soft tissue swelling at physical examination. Depicted are the joints of patients that developed arthritis. Grey bars represent unaffected joints, black bars represent joints in which arthritis developed.

One hundred and ninety-two patients with mild to moderate joint complaints were examined. Their baseline characteristics are shown in Table 1. Of these patients, 45 (23%) developed arthritis in one or more joints after a mean follow up of 11 (SD ± 9, range 1 to 41) months. Their median tender joint count (of 53 joints) at the time of arthritis development was 6 (IQR 3 to 10), while their median swollen joint count (of 44 joints) was 3 (IQR 2 to 6). Arthritis development occurred mostly in the wrist, and MCP, PIP and MTP joints (Figure 1). Of the 45 patients that developed arthritis, 22 (49%) had RA according to the 1987 ACR criteria [12], and 23 (51%) were diagnosed with undifferentiated arthritis (UA). Of these 23 patients, 11 patients later developed RA, and 9 patients never fulfilled more than 3 ACR RA criteria and remained UA patients. Three UA patients went into spontaneous remission. The presence of pain in a joint at physical examination at baseline was associated with arthritis development in that joint (OR 3.2, 95% CI 1.8 to 5.9, *P *< 0.001), with a positive predictive value of 11% and a negative predictive value of 96% (data not shown). This association was also present when only the subgroup of patients who developed arthritis was considered. Within this group the presence of pain was associated with arthritis development with an OR of 4.1 (95% CI 2.4 to 7.2, *P *< 0.001; Figure 2).

**Table 1:** Baseline characteristics

	n = 192
Age in years, mean ± SD	47 ± 11
Female sex	138 (72%)
Arthralgia duration in months, median (IQR)	12 (9-36)
Number of reported painful joints, median (IQR)	2 (0-4)
Tender joint count (53 joints), median (IQR)	0 (0-1)

Antibody status	
ACPA negative, IgM-RF positive	60 (31%)
ACPA positive, IgM-RF negative	71 (37%)
ACPA positive, IgM-RF positive	61 (32%)

ACPA, anti citrullinated protein antibodies; IQR, interquartile range; RF, rheumatoid factor; SD, standard deviation.

### Ultrasonography

#### Healthy controls

Eighty-four joints of nine healthy controls were examined. In one joint, grade 2 joint effusion was detected. In 18 joints (21%), grade 1 joint effusion was seen and in five joints (6%) grade 1 synovitis was seen. No other US abnormalities were detected.

#### Interobserver reliability

The two investigators performed double US of 148 joints of 14 patients. For the presence of joint effusion, synovitis, power Doppler signal and tenosynovitis, the interobserver agreement showed a high overall agreement (88 to 92%). However, kappa values were low for the presence of joint effusion and moderate for the presence of synovitis and power Doppler (Table 2). For tenosynovitis, kappa values could not be calculated due to very low prevalence. Dichotomization of the outcome variables did not influence interobserver reliability for joint effusion or synovitis and slightly reduced reliability for power Doppler.

**Table 2:** Interobserver reliability

	Weighted Kappa (95% CI)*	Kappa (95% CI)^†^	Overall agreement^†^
Joint effusion	0.22 (0.10-0.43)	0.23 (0.00-0.63)	92%
Synovitis	0.47 (0.23-0.70)	0.46 (0.24-0.67)	88%
Power Doppler	0.67 (0.46-0.91)	0.56 (0.34-0.77)	91%
Tenosynovitis	‡	‡	91%

* weighted kappa values were calculated for ordinal data, † kappa values and overall agreement were calculated for dichotomized data, ‡ kappa values could not be calculated, because one observer did not score any abnormalities.

CI, confidence interval.

#### US and arthritis development at joint level

US was performed on 1823 joints in total (1017 MCP, 252 PIP, 225 MTP, 316 wrist joints, 3 ankle and 10 knee joints), of which 483 were joints of patients who developed arthritis. Of the joints in which US had been performed, 78 (4.3% of the total or 16% of those with arthritis) were swollen at the time of arthritis development. In total 221 joints were swollen at the time of arthritis development. The presence of pain in a joint at physical examination at baseline was associated with the presence of joint effusion, synovitis, or power Doppler signal in that joint at baseline (OR 3.22, 4.77 and 7.08, respectively; Table 3). Moreover, the presence of joint effusion, synovitis, or power Doppler signal in a joint was associated with arthritis development in that joint (OR 3.07, 5.45 and 5.50, respectively; Table 4). Corresponding positive predictive values were 12%, 18% and 18%, respectively. The combination of the presence of synovitis and power Doppler signal increased the risk for the development of arthritis (OR 12.9) with a positive predictive value of 35%. Prevalence of other combinations was too low to calculate the corresponding risk (Table 4).

**Table 3:** Association of pain at baseline with ultrasound abnormalities at baseline

Joints with	Pain - n = 1705	Pain + n = 118	OR (95% CI)
Joint effusion grade 2-3	28 (1.6%)*	6 (5.1%)	3.22 (1.30-7.94)
Synovitis grade 2-3	29 (1.7%)	9 (7.6%)	4.77 (2.20-10.3)
Power Doppler grade1-3	34 (2.0%)^†^	15 (13%)	7.08 (3.73-13.4)

* n = 1696 (0.5% missing), †: n = 1686 (1% missing).

CI, confidence interval; OR, odds ratio.

**Table 4:** Association of ultrasound abnormalities with arthritis development at joint level

Joints with	Arthritis - n = 1745	Arthritis + n = 78	OR (95% CI)	PPV
Joint effusion grade 2-3	30 (1.7%)	4 (5.1%)	3.07 (1.05-8.94)	12%
Synovitis grade 2-3	31 (1.8%)*	7 (9%)	5.45 (2.32-12.8)	18%
PD grade 1-3	40 (2.3%)^†^	9 (11.5%)	5.50 (2.57-11.9)	18%
Synovitis and PD	11 (0.6%)	6 (7.7%)	12.9 (4.65-36.0)	35%
Joint effusion and synovitis	5 (0.3%)	0	na	
Joint effusion and PD	6 (0.3%)	0	na	

* n = 1735 (0.5% missing), † n = 1726 (1% missing), CI, confidence interval; na, not applicable due to low numbers; OR, odds ratio; PD, power Doppler; PPV, positive predictive value.

#### US and arthritis development at patient level

US was performed in 192 patients. The median number of scanned joints was 8 (IQR 6 to 10), mainly MCP, PIP and wrist joints. A positive trend was seen for the presence of joint effusion, synovitis, power Doppler signal, or tenosynovitis in one or more joints at baseline and the development of arthritis, although this did not reach statistical significance (Table 5).

**Table 5:** Association of ultrasound abnormalities with arthritis development at patient level

# patient with ≥1 joint	Arthritis -n = 147	Arthritis +n = 45	Arthritis +UA n = 12	Arthritis +RA n = 33	OR (95% CI)(Arthritis - vs. arthritis +)
Joint effusion grade 2-3	14 (10%)	8 (18%)	3 (25%)	5 (15%)	2.05 (0.80-5.27)
Synovitis grade 2-3	17 (12%)	7 (16%)	1 (8%)	5 (18%)	1.41 (0.54-3.65)
PD grade 1-3	23 (16%)	10 (22%)	1 (8%)	9 (27%)	1.54 (0.67-3.54)
Tenosynovitis grade 2-3	9 (6%)	4 (9%)	0	4 (12%)	1.50 (0.44-5.11)

CI, confidence interval; OR, odds ratio; PD, power Doppler; RA, rheumatoid arthritis; UA, undifferentiated arthritis.

## Discussion

US utility and predictive properties in ACPA and/or IgM-RF-positive arthralgia patients at risk for developing arthritis, but without clinical arthritis, were studied. One-quarter of the patients developed arthritis during follow up and in this group a trend was seen towards more US abnormalities at baseline than in the patients who did not develop arthritis. Furthermore, at the joint level a significant association of US abnormalities and the development of arthritis in that joint was found. Although the positive predictive values for arthritis development of US abnormalities were moderate and only slightly better than for pain at physical examination, the combination of synovitis and power Doppler signal increased predictive properties. Both synovitis and power Doppler signal were better predictors than the presence of joint effusion and the presence of power Doppler signal correlated better with the presence of pain than grey scale US. These results imply that US is able to detect subclinical arthritis in patients that later develop arthritis and are in line with previous studies that showed that US is more sensitive than clinical examination [5]. The advantage of power Doppler over other modalities has also been shown before. It was more predictive than grey scale US for relapse or radiographic progression in RA patients in clinical remission [13,14].

There are some limitations to this study. First, the interobserver reliability was not optimal. Although overall agreement was good, kappa values were low to moderate. This could partly be explained by the nature of the studied population in which the prevalence of US abnormalities is low and abnormalities, if present, have a low grade. A low prevalence and the difficulty of the differentiation of low-grade abnormalities from normal both decrease interobserver reliability.

A second limitation is that the joints chosen for scanning were selected based on the presence of pain at physical examination or as reported by the patient. Although this resembles a clinical setting, this selection procedure might not be optimal, because this results in different sets of joints and total joint scores cannot be compared.

Finally, at the patient level only a trend was seen towards more development of arthritis in patients that showed US abnormalities. The fact that this trend did not reach statistical significance can partly be explained by low power. The results will need to be confirmed in further studies with larger numbers of patients. On the other hand, US abnormalities were detected in patients that did not develop arthritis. These could be patients that will develop arthritis in the future or abnormalities could have been detected in patients with subclinical inflammation that subsided spontaneously. For clinical decision-making, it is important to discriminate these two patient categories from one another and thus specificity should be increased. This might be achieved by repeating US after a few months. Repeated US could also clarify whether multiple joints show US abnormalities successively in these patients.

Another improvement could be the use of a standardized US joint count with a defined set of joints. Together with regular training sessions, this would probably increase reliability [15]. A multiple joint scoring system would be more time-consuming, but when only the joints that are mostly afflicted by RA are chosen, this would save time and could nevertheless result in higher sensitivity [5]. Furthermore, if such a system is able to reliably predict arthritis development, it could be used in the clinic to support treatment decisions. Various simplified US joint count scoring systems have been evaluated in patients with early and established RA that seem feasible to assess joint inflammation in these patient groups [16,17]. Such a scoring system will have to be validated for patients presenting with arthralgia.

Alternatively, additional imaging techniques could be used to study subclinical inflammation in seropositive arthralgia patients. Magnetic resonance imaging and positron emission tomography scanning are playing an increasingly important role in the investigation and management of RA [18]. These techniques could be useful on their own or adjacent to US; their use is currently being investigated in a subgroup of the present cohort.

## Conclusions

US could become a promising diagnostic tool in patients with seropositive arthralgia. It is associated with arthritis development at the joint level and when more extensive US scoring systems are used, it might be able to predict arthritis development both at the patient and joint levels. Further research is necessary to verify this.

## Abbreviations

ACPA: anti-citrullinated protein antibodies; anti-CCP: anti-cyclic citrullinated peptide; CI: confidence interval; DMARD: disease-modifying anti-rheumatic drug; ELISA: enzyme-linked immunosorbent assay; IgM-RF: immunoglobulin-M rheumatoid factor; IQR: inter quartile range; MCP: metacarpal phalangeal; MTP: metatarsophalangeal; OR: odds ratio; PIP: proximal interphalangeal; RA: rheumatoid arthritis; SD: standard deviation; UA: undifferentiated arthritis; US: ultrasonography.

## Competing interests

The authors declare that they have no competing interests.

## Authors' contributions

This study was designed by WHB, DS and CJL. MRM and HW performed the US. All other data was collected by WHB, LAS and DS. Statistical analysis was carried out by LAS and WHB. All authors were involved in drafting the article or revising it critically for important intellectual content. All authors read and approved the final manuscript.
